# Left ventricular septal pacing versus left bundle branch pacing in the treatment of atrioventricular block

**DOI:** 10.1111/anec.12944

**Published:** 2022-03-10

**Authors:** Yu Zhou, Jinfeng Wang, Youquan Wei, Wenbo Zhang, Yuwen Yang, Shibao Rui, Changlin Ju

**Affiliations:** ^1^ 569222 Department of Emergency The First Affiliated Hospital of Wannan Medical College Wuhu Anhui China; ^2^ 569222 Department of Cardiology The First Affiliated Hospital of Wannan Medical College Wuhu Anhui China

**Keywords:** cardiac function, electrocardiogram, left bundle branch pacing, left ventricular septal pacing, physiological pacing

## Abstract

**Background:**

This study aimed to evaluate the feasibility and clinical response of LVSP as an alternative to LBBP.

**Methods:**

This was a retrospective study of pacemaker implantation, and 46 consecutive patients with pacemaker implantation were enrolled in the study. The patients were divided into the LBBP and LVSP groups. Electrocardiogram characteristics, pacing parameters, cardiac function, and safety events were assessed during implantation and 12‐month follow‐up.

**Results:**

The procedure time was significantly increased in the LBBP group compared with the LVSP group (53.52 ± 14.39 min vs. 38.13 ± 11.52 min, respectively, *p* = .000). The pacing QRS duration (PQRSD) decreased by 14.09 ± 41.80 ms in the LBBP group and increased by 9.70 ± 29.60 ms in the LVSP group (*p* = .031). Furthermore, the left ventricle activation time (LVAT) was shorter in the LBBP group than in the LVSP group (48.70 ± 13.67 ms vs. 58.70 ± 13.67 ms, *p* =  .032). During the 12‐month follow‐up, pacing thresholds remained low and stable, and there was no significant decrease in cardiac function. No adverse event was observed during the follow‐up period.

**Conclusions:**

Both LBBP and LVSP are safe and feasible methods. LVSP is a good option when multichannel electrophysiological instruments are not available and when the time available for the procedure is limited.

## Graphical abstract

Comparison of the operation time and ECG parameters between the LBBP and LVSP groups. PQRSD, paced QRS duration; QRSD, QRS duration; ΔQRSD = QRSD‐PQRSD; LVAT, left ventricle activation time. Values are mean; *p* value < .05 was considered to be statistically significant.

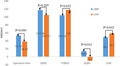

## 1 INTRODUCTION

Right ventricular apical pacing (RVAP) is widely used in clinical practice due to its low pacing threshold and simple operation. However, experimental evidence revealed that long‐term RVAP damages the function of the left ventricle, which results in increased hospitalization rate, mortality, and incidence of atrial fibrillation (Kiehl et al., 2016; Sohn et al., 2017). While there is still a debate concerning the best pacing site of the right ventricular septum (Kaye et al., 2015; Muto et al., 2018), His pacing is considered to be the most suitable pacing for the electrophysiological activation sequence. Nevertheless, its application in clinical practice is still limited due to its complex operating technology, high pacing threshold, and rapid power consumption (Vijayaraman, Chung, et al., 2018; Vijayaraman, Naperkowski, et al., 2018).

Left bundle branch pacing (LBBP) is a newly discovered physiological pacing, and its safety has been proven by many studies (Chen et al., 2019; Zang et al., 2019). LBBP can be achieved with a high success rate and low capture thresholds. Left ventricular dysfunction improved significantly during follow‐up (Ravi et al., 2020). However, LBBP, under the monitoring of multichannel electrophysiological instruments, must find P potential to accurately locate the electrode and repeatedly test the pacing threshold and impedance to prevent the electrode from penetrating the left ventricular membrane into the heart chambers and thus inducing a thrombus. Its operation takes a long time and may increase the chances of developing complications. The electrode of the left ventricular septal pacing (LVSP) is implanted in the interventricular septum, and its depth is more than half of the septum (Mills et al., 2009; Peschar et al., 2003), but with a certain distance from the left ventricular membrane surface; thus, it saves time to measure the parameters and blindly operated without a multichannel instrument.

LBBP and LVSP share similar characteristics regarding lead placement in the left septum; however, LBBP is characterized by left septal subendocardial lead placement and confirmation of left bundle branch (LBB) capture using pacing maneuvers (Huang et al., 2019). In contrast, LVSP is achievable over a larger area and with shallower lead positions (Wu et al., 2020).

Moreover, the hemodynamic effect of LVSP pacing in the acute phase is similar to that of His pacing (Salden et al., 2020). It is, therefore, worth investigating the possible short‐term safety and efficacy achieved with LVSP compared with LBBP. To this end, this article compared the clinical outcomes of LVSP and LBBP implantation in the right ventricle.

## 2 METHODS

### 2.1 Study population

All the patients from the First Affiliated Hospital of Wannan Medical College who received implantable dual‐chamber or single‐chamber pacemakers according to the established guidelines from April 2019 to December 2020 were included in this study. Inclusion and exclusion criteria: the study subjects were all single‐ or dual‐chamber pacemaker patients treated with LBBP or LVSP implantation. Two groups of patients were formed: the LBBP group (*n* = 23) included 12 men and 11 women with an average age of 72.04 years old, and the LVSP group (*n* = 23) included 11 men and 12 women with an average age of 71.08 years old.

At the time of procedures, paced QRS duration (PQRSD), QRS duration (QRSD), and left ventricle activation time (LVAT) were recorded for comparison between groups. At the one‐year follow‐up, the index of capture threshold (V), R‐wave amplitude (mV), impedance (Ω), and echocardiography, including left ventricular end‐diastolic diameter (LVEDD, mm) and left ventricular ejection fraction (LVEF, %), were analyzed. The protocol was approved by the hospital Institutional Review Board, and written informed consent was collected from all the patients.

### 2.2 Implantation procedure

#### 2.2.1 LBBP

**FIGURE 1:**
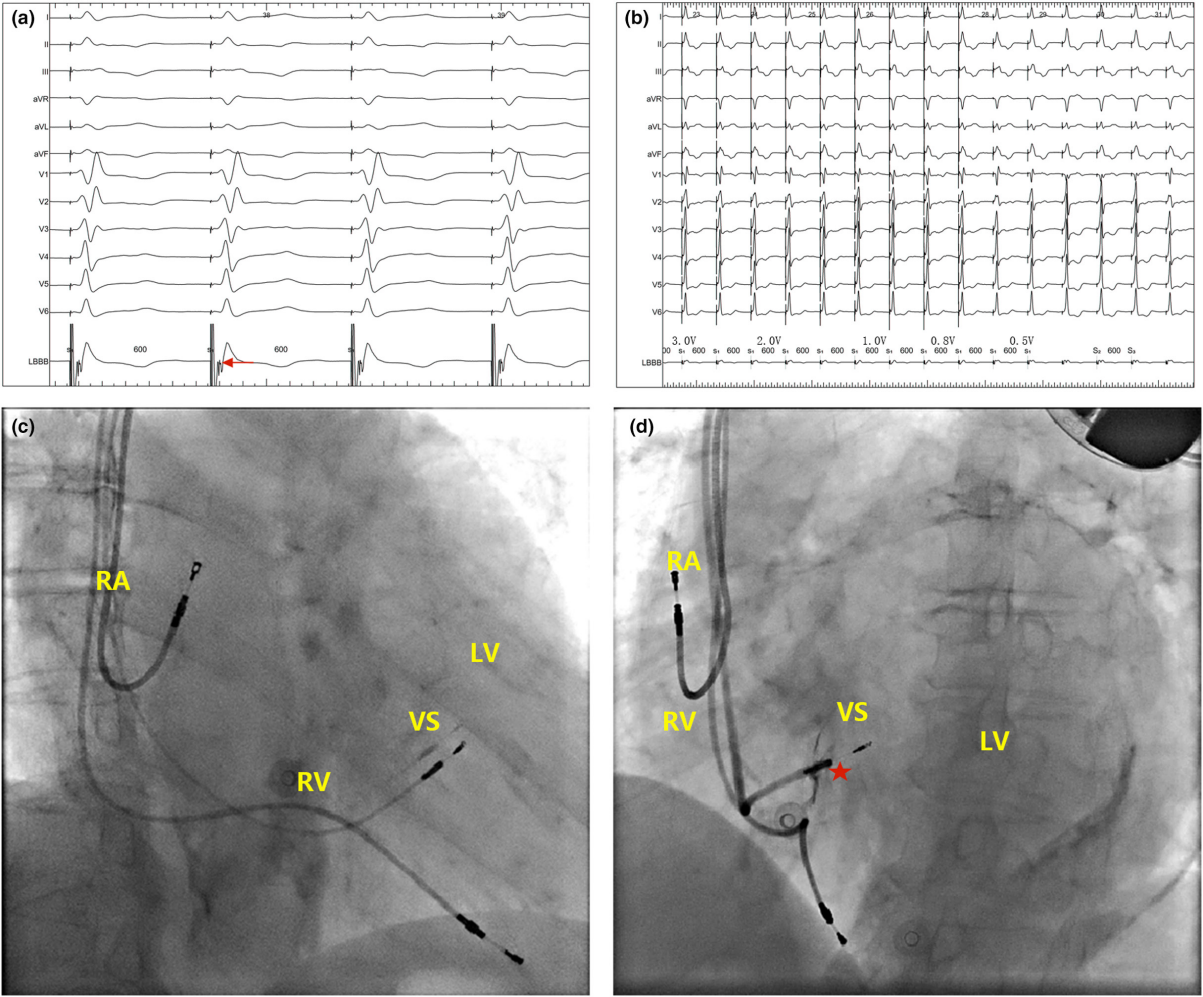
LBBP. (a) Characteristics of LBBP pattern and P potential (red arrow) in intracavitary ECG after pacing; (b) electrocardiographic changes of body surface with different pacing voltage (3–0.5 V); (c and d) implantation of 3830‐lead (red pentagram) in fluoroscopic imaging RAO 30° fluoroscopy and LAO 40° intrathecal angiography after 3830‐electrode implantation of a patient with PICM. LBBP, left bundle branch pacing; PICM, pacing‐induced cardiomyopathy

**FIGURE 2:**
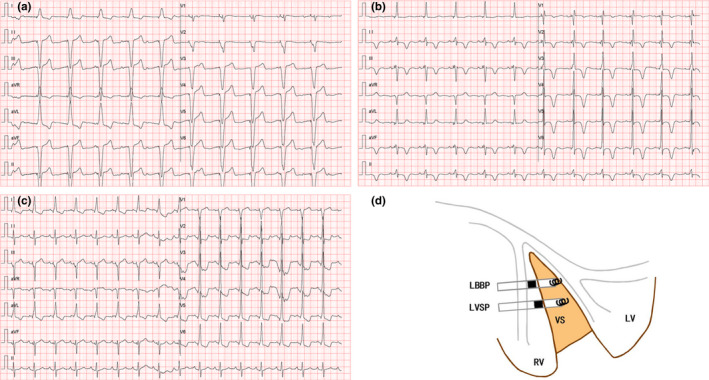
Characteristics of LBBP and LVSP in 12‐lead ECG and schematic diagram. (a) ECG in DDD mode after right ventricular apical pacing; (b) ECG after right ventricular apical pacing upgraded to LBBP; (c) ECG in DDD mode after LVSP; and (d) schematic diagram of LBBP and LVSP. LBBP, left bundle branch pacing; LVSP, left ventricular septal pacing; RV, right ventricle; LV, left ventricle; VS, ventricular septum; ECG, electrocardiogram

The implantation of the left bundle electrode was achieved following the previous work in literature (Chen & Li, 2019; Huang et al., 2019). First, the C315 delivery sheath was inserted via the axillary vein to the His bundle region in the atrioventricular septum. Next, the 3830‐pacing lead was advanced through the sheath to detect the His potential under the fluoroscopic right anterior oblique (RAO) 30°, and the image was used as a reference. The sheath with the pacing lead was further moved down 10~20 mm along the ventricular septum and created the electrocardiograph (ECG) QRS morphology of the left bundle branch block (LBBB) pattern (W‐shaped “notch” in the V1 lead) (MesbahTahaHassanin et al., 2019). Once the pacing lead was perpendicular to the right ventricular septum in the left anterior oblique (LAO) 40°, the pacing lead was rapidly rotated clockwise 4–5 turns, p tested the electrode impedance, perception, and threshold until the left bundle branch (LBB) was captured (Figure 1a,b), and then fixed the screwed depth of the left ventricular electrode by angiography (Figure 1c,d), removed the sheath, and recorded the time from puncture to the completion of the LBB electrode as the LBBP operation time, the time from the pacing pin to QRS peak as the left ventricle activation time (LVAT) in ECG V5, and the time from the pacing pin to the end of QRS as the PQRSD (Figure 2a–c).

#### 2.2.2 LVSP

The 3830‐electrode was implanted medially in the ventricular septum with a rotation depth of more than half of the septal thickness (Figure 2d). The parameters of the pacing threshold, perception, operation time, PQRSD, LVAT, and complication were recorded.

### 2.3 Statistics analysis

The SPSS software version 22.0 (IBM, Armonk, New York) was used to perform all the statistical analyses. Normally distributed continuous data were expressed as the mean ± SD, while categorical data were described as the number (%), and t or χ^2^ test was used to examine the aforementioned differences. All the tests were two‐sided, and a *p*‐value < .05 was considered to be statistically significant.

## 3 RESULTS

### 3.1 Baseline characteristics

The study cohort consisted of patients with sinus node dysfunction and AV conduction block. The left ventricular ejection fraction (LVEF) was 58.89 ± 11.93%, with four heart failure patients having a low LVEF (EF <35%) and two patients with failed cardiac resynchronization therapy/defibrillation (CRT/D). Three patients diagnosed with pacemaker‐induced cardiomyopathy (PICM) with EF <50% were upgraded to LBBP. Furthermore, 43 patients were implanted with DDD pacemakers and 3 patients were implanted with VVI pacemakers. There were no statistically significant differences in clinical baseline data between the two groups (Table 1).

**TABLE 1:** Patient baseline characteristics

Items	LBBP (*N* = 23)	LVSP (*N* = 23)	*p*‐value
Demographics			
Age (years)	72.04 ± 11.41	71.78 ± 7.08	.926
Men, *N* (%)	12 (52.17%)	11 (47.83%)	.768
Comorbidities, *N* (%)			
Hypertension	16 (69.57%)	17 (73.91%)	.743
Diabetes	2 (8.70%)	5 (21.74)	.218
Cardiomyopathy	3 (13.04%)	0(0.00%)	.076
Ischemic stroke	3 (13.04%)	4 (17.39%)	.681
Atrial fibrillation	5 (21.74%)	8 (34.78%)	.326
Indication category, *N* (%)			
Sinus node dysfunction	8 (34.78%)	8 (34.78%)	.189
AVB	12 (52.17%)	15 (65.22%)	.189
PICM	3 (13.04%)	0 (0.00%)	.189
Device, *N* (%)			
VVI	1 (4.35%)	2 (8.70%)	1.00
DDD	22 (95.65%)	21 (91.30%)	1.00

Note

Continuous data are presented as mean ± standard deviation (SD), and categorical data are presented as the number of subjects (n) and percentage (%). *p*‐value <.05 was considered to be statistically significant.

Abbreviations: AVB, atrioventricular block; PICM, pacing‐induced cardiomyopathy.

### 3.2 Implantation results

**FIGURE 3:**
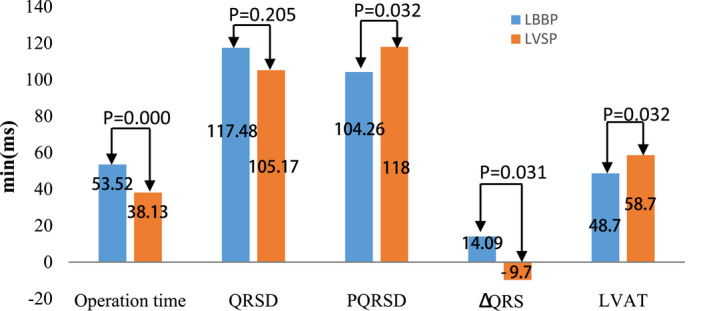
Comparison of the operation time and ECG parameters between the LBBP and LVSP groups. PQRSD, paced QRS duration; QRSD, QRS duration; ΔQRSD = QRSD‐PQRSD; LVAT, left ventricle activation time. Values are mean; *p* < .05 was considered to be statistically significant

As shown in Table 2, of the 23 patients who successfully underwent LBBP, 22 (95.65%) patients were implanted with dual‐chamber pacemakers and 1 (4.35%) with a single‐chamber pacemaker. On the contrary, of the 23 patients in the LVSP group, 21 (91.30%) were implanted with dual‐chamber pacemakers and 2 (8.70%) were implanted with single‐chamber pacemakers. The operation time was significantly longer in the LBBP group compared with the LVSP group (38.13 ± 11.52 minutes vs. 53.52 ± 14.39 minutes, respectively, *p* = .000) (Figure 3). In addition, no procedure‐related complications and adverse events were reported in either group.

**TABLE 2:** Comparison of pacing parameters and echocardiographic data of the LBBP group and the LVSP group

Parameters	Time	LBBP	LVSP	*p*‐values
Pacing parameter				
Capture threshold (V)	At implantation	0.70 ± 0.14	0.66 ± 0.18	.928
	1‐year follow‐up	0.88 ± 0.18	0.82 ± 0.19	.324
	*p* for time	.001	.004	—
R‐wave amplitude (mV)	At implantation	9.20 ± 2.11	9.89 ± 2.14	.279
	1‐year follow‐up	9.58 ± 1.90	9.43 ± 2.30	.808
	*p* for time	.96	.865	—
Impedance (Ω)	At implantation	634.57 ± 112.81	670.78 ± 137.97	.313
	1‐year follow‐up	636.83 ± 105.53	667.83 ± 146.73	.415
	*p* for time	.925	.928	—
Ultrasonic cardiogram				
LVEDD (mm)	At implantation	51.13 ± 8.22	48.78 ± 7.83	.327
	1‐year follow‐up	49.48 ± 6.85	48.91 ± 7.02	.784
	*p* for time	.463	.953	—
LVEF (%)	At implantation	59.61 ± 10.36	59.96 ± 10.14	.909
	1‐year follow‐up	58.65 ± 8.05	58.52 ± 11.32	.964
	*p* for time	.728	.653	—

Note

*p*‐value <.05 was considered statistically significant.

Abbreviations: LBBP, left bundle branch pacing; LVEDD, left ventricular end‐diastolic diameter; LVEF, left ventricular ejection fraction; LVSP, left ventricular septal pacing.

### 3.3 Pacing parameters and cardiac function

Ventricular lead parameters, that is, the capture threshold, R‐wave amplitude, and ventricular impedance, did not differ significantly between the two groups during the procedure and at the 1‐year follow‐up. Although the pacing thresholds in both groups increased slightly from 0.70 ± 0.14 V to 0.88 ± 0.18 V (*p* = .001) in LBBP group and from 0.66 ± 0.18 V to 0.82 ± 0.19 V (*p *= .004), they were still in the lower and safe range. Similarly, LVEDD and LVEF did not change significantly between the two groups of patients at the preoperative and one‐year postoperative follow‐up (*p* .05) (Table 2).

### 3.4 ECG characteristics

Compared with the ECG parameters before and after pacing, there was no significant difference in the QRSD before pacing between the two groups. After the procedure, the PQRSD decreased by 14.09 ± 41.80 ms in the LBBP group and increased by 9.70 ± 29.60 ms in the LVSP group, with a significant difference in the changes (104.26 ± 19.00 ms vs. 118.09 ± 23.20 ms, respectively, *p* =  .032). Furthermore, the activation time of the left ventricle was shorter in the LBBP group than in the LVSP group (48.70 ± 13.67 ms vs. 58.70 ± 13.67 ms, respectively, *p* = .032) (Table 3, Figure 3).

**TABLE 3:** Comparison of pacing parameters and echocardiographic data of the LBBP group and the LVSP group

Parameters	Time	LBBP	LVSP	*p*‐values
Operating time (min)	At implantation	53.52 ± 14.39	38.13 ± 11.52	.000
PQRSD (ms)	At implantation	104.26 ± 19.00	118.09 ± 23.20	.032
QRSD (ms)	At implantation	117.48 ± 36.70	105.17 ± 27.56	.205
ΔQRSD (ms)	At implantation	14.09 ± 41.80	−9.70 ± 29.60	.031
LVAT (ms)	At implantation	48.70 ± 13.67	58.70 ± 13.67	.032
Capture threshold (volts)	At implantation	0.70 ± 0.14	0.66 ± 0.18	.928
	1‐year follow‐up	0.88 ± 0.18	0.82 ± 0.19	.324
	*p* for time	.001	.004	—
R wave amplitude (mV)	At implantation	9.20 ± 2.11	9.89 ± 2.14	.279
	1‐year follow‐up	9.58 ± 1.90	9.43 ± 2.30	.808
	*p* for time	.960	.865	—
Impedance (ohms)	At implantation	634.57 ± 112.81	670.78 ± 137.97	.313
	1‐year follow‐up	636.83 ± 105.53	667.83 ± 146.73	.415
	*p* for time	.925	.928	—

Note

Values are mean (SD), *p*‐value < .05 was considered to be statistically significant.

Abbreviations: LVAT, left ventricle activation time; PQRSD, paced QRS duration; QRSD, QRS duration; ΔQRSD = QRSD‐PQRSD.

## 4 DISCUSSION

Our study demonstrated the LBBP and LVSP pacing methods are both safe and effective. Compared with LVSP, LBBP is more physiologically compatible as it shortens the QRS duration and activates the left ventricle earlier, but LVSP is a good option when critical equipment is lacking or time is limited.

The feasibility of LVSP has been proven by a series of experiments from basic research to clinical practice. In 1970, Dirk Durer mapped the human heart in vitro and pointed out that the earliest exciting site in the ventricle is the left ventricular septum (Durrer et al., 1970). Therefore, the best pacing site should be the closest one to the normal ventricular activation after pacing. After that, a series of animal and clinical experimental studies on the left ventricular septum pacing were carried out. In 2002, Grosfeld firstly confirmed the feasibility of LVSP in animal experiments (Grosfeld et al., 2002). Later in 2009, Mills showed that RVAP or right ventricular septal pacing (RVSP) significantly increased the asynchrony of the ventricular electrical activity, while left ventricular apical pacing (LVAP) or LVSP resulted in less difference in the asynchrony mechanical work and blood flow redistribution, which maintained the normal systolic and diastolic function of the ventricle (Mills et al., 2009). In 2016, Mafi‐Rad conducted a prospective study of LVSP in patients with sick sinus syndrome (SSS) (Mafi‐Rad et al., 2016). The results showed that the PQRSD of LVSP (144 ± 20 ms) was significantly shorter than that of RVSP (165 ± 17 ms, *p* = .004) and RVAP (172 ± 33 ms, *p* = .002). The capture threshold (0.5 ± 0.2 V) and R‐wave amplitude (12.2 ± 6.7 mv) of the pacing parameters were stable for 6 months after the operation. In the same year, LVSP research conducted by Leonard on patients with complete LBBB (CLBBB) showed that LVSP could improve the left ventricular systolic function by 10% and reduce the dispersion of ventricular repolarization by 30% (Rademakers et al., 2016).

The previous experiments have proven that LVSP is safe and effective for SSS and atrioventricular block (AVB) patients. In this study, LVSP was successfully implanted in all the patients without complications. The postoperative pacemaker parameters were very ideal, with a PQRSD of only 118.09 ± 23.20 ms, which was significantly lower than the value reported in the study by Masih (144 ± 20 ms). The difference between these two values may be related to the position and depth of the implanted electrode.

LBBP is a creative development based on LVSP and His pacing. In 2017, Huang reported that a patient with CRT failure underwent LVSP treatment and confirmed that the pacing electrode was successfully implanted into the LBB; the threshold (0.5 V, 0.5 ms) pacing could capture LBB and correct CLBBB. After 1‐year of follow‐up, patients had a stable pacing threshold, an increase in EF from 32% to 62%, and a decrease in left ventricular end‐diastolic diameter (LVEDD) from 76 mm to 42 mm (Huang et al., 2017). This shows that LVSP can selectively lower the threshold value of pacing the distal part of LBB across the pathological part of LBB and achieve physiological pacing similar to that of His bundle pacing (Sharma et al., 2017). Since then, the safety of LBBP has been confirmed by the research performed at several single centers in China (Chen et al., 2019; Gao et al., 2020; Guo et al., 2020). This study showed that LBBP pacing could shorten the QRS time and activate the ventricles earlier than LVSP, more physiological pacing. Because of this feature, LBBP was used as an escalation therapy for three patients with PICM.

LBBP combines the common advantages of LVSP and His pacing and compensates for their shortcomings. Although His pacing is the most physiologic one, its clinical application is limited by its shortcomings such as low perception, high capture threshold, and operational complexity. In particular, the progression of His bundle lesions distally poses a potential problem for His pacemaker therapy in AVB patients (Vijayaraman, Chung, et al., 2018; Vijayaraman, Naperkowski, et al., 2018), often requiring the implantation of apical pacemaker backup electrodes. Although LBBP is more complex than LVSP, the observations in this study showed that LBBP has similarly good perception and threshold to those of LVSP with no pacemaker‐related infection, lead dislocation, pacing dysfunction, or other adverse events such as LVSP and better ventricular synchrony. Some other limitations of this study are as follows: 1. since it is not a random, controlled, multicenter prospective study, LBBP is not representative. 2. LVSP at different sites of the interventricular septum may affect pacing parameters and cardiac function. However, we implanted from the right ventricular septum, the location was not precise and the depth of implantation was not fixed. 3. The follow‐up time is not long enough, and the effect of LVSP on cardiac function and safety needs to be further evaluated. 4. So far, the clinical trials related to LBBP are single‐center studies and the experimental results are still lack representativeness.

## 5 CONCLUSION

Both LBBP and LVSP are safe and feasible approaches. LBBP is consistent with physiological pacing, but LVSP is a good option in the absence of electrophysiological multichannel instruments and in cases where procedure time is limited.

## 6 ETHICS APPROVAL AND CONSENT TO PARTICIPATE

This study was approved by the ethics committee of the First Affiliated Hospital of Wannan Medical College. Written informed consent was obtained from all patients.

## 7 CONSENT FOR PUBLICATION

Informed consent for the publication of the report was obtained from the patient and study participants in written form.

## CONFLICT OF INTERESTS

The authors declare that they have no competing interests.

## AUTHORS’ CONTRIBUTIONS

YZ, the first author, wrote the manuscript. JFW, YQW, YWY, SBR, and CLJ performed the pacemaker implantation. All authors read and approved the final manuscript. All authors agreed to their contributions.

## DATA AVAILABILITY STATEMENT

Data are available from the corresponding author upon reasonable request due to privacy or other restrictions.

## References

[anec12944-bib-0001] Chen, K. , & Li, Y. (2019). How to implant left bundle branch pacing lead in routine clinical practice. Journal of Cardiovascular Electrophysiology, 30, 2569–2577. 10.1111/jce.14190 31535747

[anec12944-bib-0002] Chen, K. , Li, Y. , Dai, Y. , Sun, Q. , Luo, B. , Li, C. , & Zhang, S. (2019). Comparison of electrocardiogram characteristics and pacing parameters between left bundle branch pacing and right ventricular pacing in patients receiving pacemaker therapy. Europace, 21, 673–680. 10.1093/europace/euy252 30462207

[anec12944-bib-0003] Durrer, D. , van Dam, R. T. , Freud, G. E. , Janse, M. J. , Meijler, F. L. , & Arzbaecher, R. C. (1970). Total excitation of the isolated human heart. Circulation, 41, 899–912. 10.1161/01.CIR.41.6.899 5482907

[anec12944-bib-0004] Gao, M. Y. , Tian, Y. , Shi, L. , Wang, Y. J. , Xie, B. Q. , Qi, J. , Zeng, L. J. , Li, X. X. , Yang, X. C. , & Liu, X. P. (2020). Electrocardiographic morphology during left bundle branch area pacing: Characteristics, underlying mechanisms, and clinical implications. Pacing and Clinical Electrophysiology, 43, 297–307. 10.1111/pace.13884 32045008

[anec12944-bib-0005] Grosfeld, M. J. , Res, J. C. , Vos, D. H. , de Boer, T. J. , & Bos, H. J. (2002). Testing a new mechanism for left interventricular septal pacing: The transseptal route; a feasibility and safety study. Europace, 4, 439–444. 10.1053/eupc.2002.0253 12408265

[anec12944-bib-0006] Guo, J. , Li, L. , Meng, F. , Su, M. , Huang, X. , Chen, S. , Li, Q. , Chang, D. , & Cai, B. (2020). Short‐term and intermediate‐term performance and safety of left bundle branch pacing. Journal of Cardiovascular Electrophysiology, 31, 1472–1481. 10.1111/jce.14463 32239598 PMC7317583

[anec12944-bib-0007] Huang, W. , Chen, X. , Su, L. , Wu, S. , Xia, X. , & Vijayaraman, P. (2019). A beginner's guide to permanent left bundle branch pacing. Heart Rhythm: The Official Journal of the Heart Rhythm Society, 16, 1791–1796. 10.1016/j.hrthm.2019.06.016 31233818

[anec12944-bib-0008] Huang, W. , Su, L. , Wu, S. , Xu, L. , Xiao, F. , Zhou, X. , & Ellenbogen, K. A. (2017). A novel pacing strategy with low and stable output: Pacing the left bundle branch immediately beyond the conduction block. Canadian Journal of Cardiology, 33, 1736.e1‐.e3. 10.1016/j.cjca.2017.09.013 29173611

[anec12944-bib-0009] Kaye, G. C. , Linker, N. J. , Marwick, T. H. , Pollock, L. , Graham, L. , Pouliot, E. , Poloniecki, J. , Gammage, M. , Kaye, G. , Martin, P. , Pepper, C. , Tang, K. , Crozier, I. , Goode, G. , Young, G. , Petkar, S. , Langford, E. D. , Linker, N. J. , Rozkovec, A. , … McGavigan, A. (2015). Effect of right ventricular pacing lead site on left ventricular function in patients with high‐grade atrioventricular block: Results of the Protect‐Pace study. European Heart Journal, 36, 856–862. 10.1093/eurheartj/ehu304 25189602

[anec12944-bib-0010] Kiehl, E. L. , Makki, T. , Kumar, R. , Gumber, D. , Kwon, D. H. , Rickard, J. W. , Kanj, M. , Wazni, O. M. , Saliba, W. I. , Varma, N. , Wilkoff, B. L. , & Cantillon, D. J. (2016). Incidence and predictors of right ventricular pacing‐induced cardiomyopathy in patients with complete atrioventricular block and preserved left ventricular systolic function. Heart Rhythm: The Official Journal of the Heart Rhythm Society, 13, 2272–2278. 10.1016/j.hrthm.2016.09.027 27855853

[anec12944-bib-0011] Mafi‐Rad, M. , Luermans, J. G. L. M. , Blaauw, Y. , Janssen, M. , Crijns, H. J. , Prinzen, F. W. , & Vernooy, K. (2016). Feasibility and acute hemodynamic effect of left ventricular septal pacing by transvenous approach through the interventricular septum. Circulation: Arrhythmia and Electrophysiology, 9, e003344. 10.1161/CIRCEP.115.003344 26888445

[anec12944-bib-0012] MesbahTahaHassanin, M. , ShafieAmmar, A. , Abdullah, R. M. , & Khedr, M. H. (2019). Different lead locations guided by fluoroscopy and ECG parameters and their effect on patients functional status. The Egyptian Heart Journal, 71, 29. 10.1186/s43044-019-0023-1 31782052 PMC6883006

[anec12944-bib-0013] Mills, R. W. , Cornelussen, R. N. , Mulligan, L. J. , Strik, M. , Rademakers, L. M. , Skadsberg, N. D. , van Hunnik, A. , Kuiper, M. , Lampert, A. , Delhaas, T. , & Prinzen, F. W. (2009). Left ventricular septal and left ventricular apical pacing chronically maintain cardiac contractile coordination, pump function and efficiency. Circulation: Arrhythmia and Electrophysiology, 2, 571–579. 10.1161/CIRCEP.109.882910 19843926

[anec12944-bib-0014] Muto, C. , Calvi, V. , Botto, G. L. , Pecora, D. , Porcelli, D. , Costa, A. , Ciaramitaro, G. , Airò Farulla, R. , Rago, A. , Calvanese, R. , Baratto, M. T. , Reggiani, A. , Giammaria, M. , Patané, S. , Campari, M. , Valsecchi, S. , & Maglia, G. (2018). Chronic apical and nonapical right ventricular pacing in patients with high‐grade atrioventricular block: Results of the right pace study. BioMed Research International, 2018, 1404659. 10.1155/2018/1404659 29951525 PMC5987304

[anec12944-bib-0015] Peschar, M. , de Swart, H. , Michels, K. J. , Reneman, R. S. , & Prinzen, F. W. (2003). Left ventricular septal and apex pacing for optimal pump function in canine hearts. Journal of the American College of Cardiology, 41, 1218–1226. 10.1016/S0735-1097(03)00091-3 12679225

[anec12944-bib-0016] Rademakers, L. M. , van Hunnik, A. , Kuiper, M. , Vernooy, K. , van Gelder, B. , Bracke, F. A. , & Prinzen, F. W. (2016). A possible role for pacing the left ventricular septum in cardiac resynchronization therapy. JACC Clinical Electrophysiology, 2, 413–422. 29759859 10.1016/j.jacep.2016.01.010

[anec12944-bib-0017] Ravi, V. , Hanifin, J. L. , Larsen, T. , Huang, H. D. , Trohman, R. G. , & Sharma, P. S. (2020). Pros and cons of left bundle branch pacing: A single‐center experience. Circulation: Arrhythmia and Electrophysiology, 13, e008874. 10.1161/CIRCEP.120.008874 33198496

[anec12944-bib-0018] Salden, F. C. W. M. , Luermans, J. G. L. M. , Westra, S. W. , Weijs, B. , Engels, E. B. , Heckman, L. I. B. , Lamerichs, L. J. M. , Janssen, M. H. G. , Clerx, K. J. H. , Cornelussen, R. , Ghosh, S. , Prinzen, F. W. , & Vernooy, K. (2020). Short‐term hemodynamic and electrophysiological effects of cardiac resynchronization by left ventricular septal pacing. Journal of the American College of Cardiology, 75, 347–359. 32000945 10.1016/j.jacc.2019.11.040

[anec12944-bib-0019] Sharma, P. S. , Ellison, K. , Patel, H. N. , & Trohman, R. G. (2017). Overcoming left bundle branch block by permanent His bundle pacing: Evidence of longitudinal dissociation in the His via recordings from a permanent pacing lead. HeartRhythm Case Reports, 3, 499–502. 10.1016/j.hrcr.2017.08.002 29387538 PMC5778095

[anec12944-bib-0020] Sohn, J. , Lee, Y. S. , Park, H. S. , Han, S. , & Kim, Y. N. (2017). Predictors of an adverse clinical outcome in patients with long‐term right ventricular apical pacing. Journal of Cardiology, 70, 420–424. 10.1016/j.jjcc.2017.04.008 28551356

[anec12944-bib-0021] Vijayaraman, P. , Chung, M. K. , Dandamudi, G. , Upadhyay, G. A. , Krishnan, K. , Crossley, G. , Bova Campbell, K. , Lee, B. K. , Refaat, M. M. , Saksena, S. , Fisher, J. D. , & Lakkireddy, D. ; ACC’s Electrophysiology Council . (2018). His bundle pacing. Journal of the American College of Cardiology, 72, 927–947. 10.1016/j.jacc.2018.06.017 30115232

[anec12944-bib-0022] Vijayaraman, P. , Naperkowski, A. , Subzposh, F. A. , Abdelrahman, M. , Sharma, P. S. , Oren, J. W. , Dandamudi, G. , & Ellenbogen, K. A. (2018). Permanent His‐bundle pacing: Long‐term lead performance and clinical outcomes. Heart Rhythm: The Official Journal of the Heart Rhythm Society, 15, 696–702. 10.1016/j.hrthm.2017.12.022 29274474

[anec12944-bib-0023] Wu, S. , Sharma, P. S. , & Huang, W. (2020). Novel left ventricular cardiac synchronization: Left ventricular septal pacing or left bundle branch pacing? Europace, 22, ii10‐ii18. 10.1093/europace/euaa297 33370804

[anec12944-bib-0024] Zang, J. , Wang, Z. , Cheng, L. , Zu, L. , Liang, Z. , Hang, F. , Wang, X. , Li, X. , Su, R. , Du, J. , & Wu, Y. (2019). Immediate clinical outcomes of left bundle branch area pacing vs conventional right ventricular pacing. Clinical Cardiology, 42, 768–773. 10.1002/clc.23215 31184785 PMC6671779

